# The Role of Cannabidiol in Liver Disease: A Systemic Review

**DOI:** 10.3390/ijms25042370

**Published:** 2024-02-17

**Authors:** Si Chen, Jeon-Kyung Kim

**Affiliations:** 1 Department of Biochemistry and Molecular Biology, Jeonbuk National University Medical School, Jeonju 54896, Republic of Korea; chensi0323@naver.com; 2 Institute of New Drug Development, School of Pharmacy, Jeonbuk National University, Jeonju 54896, Jeonbuk, Republic of Korea

**Keywords:** CBD, liver disease, cannabinoid receptors, clinical trials

## Abstract

Cannabidiol (CBD), a non-psychoactive phytocannabinoid abundant in *Cannabis sativa*, has gained considerable attention for its anti-inflammatory, antioxidant, analgesic, and neuroprotective properties. It exhibits the potential to prevent or slow the progression of various diseases, ranging from malignant tumors and viral infections to neurodegenerative disorders and ischemic diseases. Metabolic dysfunction-associated steatotic liver disease (MASLD), formerly known as non-alcoholic fatty liver disease (NAFLD), alcoholic liver disease, and viral hepatitis stand as prominent causes of morbidity and mortality in chronic liver diseases globally. The literature has substantiated CBD’s potential therapeutic effects across diverse liver diseases in in vivo and in vitro models. However, the precise mechanism of action remains elusive, and an absence of evidence hinders its translation into clinical practice. This comprehensive review emphasizes the wealth of data linking CBD to liver diseases. Importantly, we delve into a detailed discussion of the receptors through which CBD might exert its effects, including cannabinoid receptors, CB1 and CB2, peroxisome proliferator-activated receptors (PPARs), G protein-coupled receptor 55 (GPR55), transient receptor potential channels (TRPs), and their intricate connections with liver diseases. In conclusion, we address new questions that warrant further investigation in this evolving field.

## 1. Introduction

Liver diseases result in two million deaths, accounting for 4% of global mortality [1]. Currently, liver disease is the 11th leading cause of death [2]. The primary causes of death are complications arising from cirrhosis and hepatocellular carcinoma (HCC). Cirrhosis typically arises from viral hepatitis, excessive alcohol consumption, and metabolic dysfunction-related steatotic liver disease (MASLD), previously referred to as non-alcoholic fatty liver disease (NAFLD). Viral hepatitis is the predominant cause of most cases of acute hepatitis, but drug-induced liver injury is increasingly contributing to the overall cases.

Cannabidiol (CBD) is one of the cannabinoids that possesses substantial therapeutic potential among numerous non-psychoactive compounds. This is substantiated by preclinical studies that have confirmed the existence of antioxidant, neuroprotective, cardioprotective, anxiolytic, and anti-inflammatory properties [3,4,5]. The study of the pharmacological effects of CBD has been growing in recent years, leading to the gradual discovery of new and exciting findings. However, clinical trials involving CBD and monitoring its effects are limited. Therefore, in the present review, we first summarized the available findings of CBD used in the treatment of different kinds of liver diseases, including MASLD, alcoholic liver disease, liver fibrosis, HCC, chemical liver injury, viral infection, autoimmune hepatitis, and liver ischemia-reperfusion injury. Additionally, we then listed the possible underlying signaling mediated by CBD. All these data indicate that CBD is a promising agent for the treatment of liver disease. More and more evidence shows that cannabinoid receptor 1 (CB1) is a key mediator of insulin resistance and liver lipogenesis. CBD acts as a negative allosteric modulator of CB1, making it a promising therapeutic agent for liver diseases [6]. The activation of cannabinoid receptor 2 (CB2) in the liver can improve liver fibrosis caused by bile duct ligation and promote liver regeneration [7]. In addition, CB2 can inhibit the deterioration of HCC [8]. CBD exhibits lower affinity towards CB1 and CB2 receptors. It appears to exert its effects more effectively through other receptors such as transient receptor potential channels (TRPs), peroxisome proliferator-activated receptors (PPARs), and G protein-coupled receptor 55 (GPR55). In chronic alcohol-fed mouse models, CBD restored liver peroxisome proliferator-activated receptor α(PPARα) protein expression and improved alcohol-induced liver metabolic imbalance and steatosis [9]. Transient receptor potential vanilloid 1(TRPV1), transient receptor potential ankyrin 1(TRPA1), and transient receptor potential melastatin 8(TRPM8) increase glucose utilization and prevent lipid accumulation and insulin resistance in the liver [10]. CBD’s ability to promote transient receptor potential vanilloid 2(TRPV2) receptor activation enhances the anticancer effects of doxorubicin in liver cancer cell lines [11]. Clinical trials on CBD in liver diseases are still lacking and often lack the scientific rigor, controls, or sample size necessary to draw clinically meaningful conclusions. More exploration in this area will be needed in the future.

## 2. CBD and Liver Disease: Molecular Mechanisms

### 2.1. Hepatic Steatosis

MASLD has rapidly become an epidemic, causing significant harm to global human health, particularly in association with obesity and related metabolic syndrome. This chronic inflammatory liver disease, closely linked to metabolic syndrome, necessitates new therapeutic approaches to enhance prevention and treatment.

Current research suggests a positive role for CBD in ameliorating hepatic metabolic imbalance and steatosis in non-alcoholic fatty liver disease. In this study, CBD treatment-induced changes in the levels of multiple lipid metabolism-related proteins in liver cells, and in vivo experiments confirmed that CBD increased lipid metabolism and mitochondrial activity, thereby reducing hepatic fat accumulation in both zebrafish and obese mice [12]. The specific mechanisms by which CBD reduces hepatic fat accumulation remain unclear; however, this study suggests that the activation of NLR family pyrin domain containing 3 (NLRP3) inflammasome occurs in MASLD, and CBD inhibits the nuclear factor kappa-light-chain-enhancer of activated B cells (NF-κB) activation, thereby suppressing the activation of macrophage NLRP3 inflammatory bodies, preventing the development of MASLD [13,14]. 

Additionally, the endocannabinoid system is closely related to metabolic disorders, and phytocannabinoids are considered a promising avenue for treating MASLD [15]. Also, CBD derivatives have lowered low-density lipoprotein levels in high-fat diet (HFD) fed mice [16].

Further research is needed to determine whether CBD can improve hepatic lipid metabolism disorders by directly interacting with endocannabinoid receptors.

CBD’s role in alleviating alcohol-induced hepatic metabolic disorders is evident in animal models. CBD protects the liver from alcohol-induced oxidative stress-induced steatosis. Also, it protects against acute alcohol-induced liver fat degeneration by inhibiting the JNK-MAPK signaling pathway and increasing autophagy levels [17]. As a result, reducing inflammatory factors and oxidative stress levels restores the expression of lipid metabolism factors, thereby decreasing alcohol-induced triglyceride and lipid accumulation and providing a specific therapeutic effect on chronic alcohol-induced hepatic metabolic disorders and steatosis [9]. Moreover, in a model of ethanol-fed mice with a high-cholesterol diet, CBD’s protective effect on the liver is reflected in regulating the inflammatory pathway NF-κB-NLRP3, as discussed earlier [18].

More and more studies suggest a close relationship between the occurrence of fatty liver disease and changes in the gut microbiota [19,20,21]. This study found that after feeding mice with HFD supplemented with CBD 2.39 mg/kg for 6 weeks, CBD reduced liver inflammation, improved glucose tolerance, and alleviated gut microbiota imbalance caused by HFD. CBD increased the abundance of *Lactobacillaceae* in the intestines of HFD-fed mice [22]. *Lactobacillaceae* produces short-chain fatty acids, a novel and feasible approach to alleviating non-alcoholic steatohepatitis (NASH) [23]. However, another study presented contradictory results. After being fed HFD for 6 weeks, mice were gavaged with CBD-rich extract at a 5 mg/kg dose every three days. This increased liver inflammation-related gene expression and decreased microbial diversity [24]. These conflicting findings raise several issues for discussion. For one, the impact of a single extracted ingredient on the composition of the gut microbiome may be different than that of extracts with multiple ingredients. Additionally, the mode of administration, concentration, and duration of administration are also factors to consider.

These research findings indicate that CBD affects hepatic lipid metabolism through multiple mechanisms, thereby exerting therapeutic effects on fatty liver disease.

### 2.2. Liver Fibrosis

In the context of liver damage, oxidative stress emerges as a primary pathogenic factor driving the progression of liver fibrosis. CBD, through various mechanisms, modulates the formation of new fibrous tissues in the liver. In this study, the metabolic regulatory characteristics of CBD were validated both in vivo and in vitro within a liver fibrosis model [9]. In a mouse model of non-alcoholic liver fibrosis induced by CCl_4_, CBD reduced the infiltration of T cells and macrophages, impeded transforming growth factor-β (TGF-β) and IL-4-induced migration of fibroblasts, and suppressed the transcription synthesis of collagen genes, thereby significantly mitigating liver fibrosis [25]. In a mouse liver fibrosis model induced by perfluorooctanesulfonic acid, CBD alleviated liver inflammation and fibrosis by regulating the formation of macrophage extracellular traps and the CCDC25-ILK-NF-κB signaling axis [26].

Activating cannabinoid receptors reduces the release of pro-inflammatory factors and oxidative stress and diminishes liver fibrosis [27]. CBD serves as an agonist for adenosine A2A/equilibrium nucleoside transporter receptors, peroxisome proliferator-activated receptor γ (PPAR γ), transient receptor potential vanilloid-type channels, and an antagonist for GPR55 receptors, and downregulates pro-inflammatory and pro-fibrotic chemokines/cytokines [28]. Further exploration is warranted to determine whether CBD positively influences liver fibrosis through endocannabinoid receptors.

The activation of hepatic stellate cells (HSCs) is considered a pivotal cellular event in response to liver injury, leading to the excessive production of type I collagen and subsequent liver fibrosis. This study demonstrates that CBD, independently of cannabinoid receptors, induces apoptosis in activated HSCs by activating the IRE1/ASK1/c-Jun N-terminal kinase pathway [29]. Additionally, CBD reduces lipid accumulation, stimulates autophagy, regulates inflammation, decreases oxidative stress, and induces apoptosis, thus reducing alcohol-related fat deposition and fibrosis in the liver [30].

### 2.3. Complications of Liver Fibrosis

CBD also plays a role in various complications of liver fibrosis. In a mouse model of hepatic encephalopathy induced by bile duct ligation, CBD increased the expression of neurotrophic factors, thereby improving cognitive and motor abilities [31]. On the other hand, CBD administration in a rat model has been shown to improve endothelial function and reduce pulmonary arterial pressure, attributed to enhanced vasodilatory response and increased endocannabinoids [32,33]. Further exploration is needed to determine whether CBD has a positive effect on the elevated portal vein pressure caused by cirrhosis. Furthermore, in this study, a novel CBD formulation alleviated angiotensin II-induced fibrosis in the mouse heart and other organs [34].

### 2.4. Hepatocellular Carcinoma and Metastasis

Phytocannabinoids and substances that elevate endogenous cannabinoid levels are considered potential drugs for the treatment of HCC [35]. The use of cannabinoids has been linked to a 55% reduction in the likelihood of developing HCC [36], with CBD demonstrating higher safety compared to other cannabinoids [37].

CBD induces apoptosis in tumor cells, inhibits cancer cell invasion and metastasis, suppresses angiogenesis, and exerts immunomodulatory effects [38]. This study found that low doses of CBD extracellular vesicles significantly reduced the viability of liver cancer cell lines in a time- and dose-dependent manner, while high doses induced cell death by activating the mitochondrial-dependent apoptotic signaling pathway, blocking the G0/G1 phase of the cell cycle [39]. CBD promotes apoptosis in cholangiocarcinoma cells, inhibits mitosis, and enhances autophagic cell death in cancer cells [40]. Additionally, CBD regulates mitochondrial function, STAT3, and prohibitin, significantly reducing the release of extracellular vesicles and microvesicles from liver cancer cell lines, thereby inhibiting cancer cell growth in vivo and increasing sensitivity to chemotherapy [41]. This study discovered that CBD forms stable hydrophobic and hydrogen bond interactions within the catalytic domain of Akt-2, suggesting that CBD’s anti-angiogenic, pro-apoptotic, cell cycle arrest, and anti-inflammatory effects in liver cancer animal models may be associated with Akt. Further exploration is needed to determine whether CBD mediates the anti-hepatocellular carcinoma effects through the PI3K-Akt pathway [42].

Combining CBD with anticancer drugs enhances the efficacy of certain anticancer drugs [43]. Under exposure to chemotherapy drugs, pyroptosis in cancer cells may progress faster than apoptosis. This study suggests that CBD induces pyroptosis in liver cancer cells in a caspase-3/gasdermin E (GSDME)-dependent manner and can inhibit aerobic glycolysis by regulating the ATF4-IGFBP1-Akt axis [44]. The combined use of CBD and cabozantinib has a better therapeutic effect on hepatocellular carcinoma, and the apoptotic effect of combination therapy is mainly related to endoplasmic reticulum stress-regulated P53 phosphorylation [45]. CBD enhances the action of doxorubicin in hepatocellular carcinoma by promoting doxorubicin entry into cancer cells through TRPV2 and inhibiting P-glycoprotein ATPase transporter to reduce doxorubicin clearance [11].

The migration and adhesion of tumor cells are fundamental characteristics of tumor metastasis. As an inhibitor of GPR55, CBD significantly reduces the adhesion and migration of colorectal cancer cells to endothelial cells and reduces the metastasis of cancer cells in the liver in a mouse colorectal cancer metastasis model [46].

### 2.5. Viral Infection

CBD has shown preventive and therapeutic effects against infections caused by HIV, SARS-CoV-2, COVID-19, and KSHV [47,48,49,50,51,52]. The current research on CBD’s role in viral hepatitis is still limited. This study found that CBD inhibits the replication of the hepatitis C virus in a dose-dependent manner while showing no activity against hepatitis B infection [53]. In approximately 75–85% of patients with acute HCV infection, the virus persists and develops into chronic hepatitis [54]. Over the past few decades, the molecular mechanisms of viral persistence and the risk factors for developing advanced liver disease and HCC have been the research focus. The focus is on the interaction between chronic infection and intracellular signal transduction and metabolic pathways (emphasizing lipid metabolism). Unfortunately, the mechanism of CBD’s involvement in liver fibrosis caused by the hepatitis C virus is not yet clear.

### 2.6. Chemical Liver Injury

CBD’s protective effect on the liver against chemical damage primarily stems from its ability to inhibit oxidative stress and apoptosis. In a CCl_4_-induced hepatic toxicity model, CBD significantly reduced the infiltration of T cells and macrophages [25]. CBD also mitigated liver damage induced by perfluorooctanesulfonic acid [26]. CBD safeguards the liver from the toxic effects of cadmium by inhibiting hepatic lipid peroxidation, preventing the depletion of reduced glutathione and nitric oxide, and inhibiting hydrogen peroxide enzyme activity [55]. Also, CBD alleviated acute liver inflammation and injury induced by cocaine and prevented seizures caused by cocaine toxicity [56]. CBD-rich mustard extracts can inhibit oxidative stress, gluconeogenesis, and hepatic lipid accumulation in iron-mediated oxidative liver damage while regulating cholinergic and purinergic activities [57].

### 2.7. Autoimmune Hepatitis

Autoimmune hepatitis is an inflammatory liver disease caused by activated T cells and macrophages. This study indicates that the use of CBD alleviates extrahepatic symptoms in patients with autoimmune hepatitis [58]. In a mouse model of acute hepatitis induced by Concanavalin A injection, CBD inhibits immune liver damage in an arginase-dependent manner by activating TRPV1 receptors and inducing bone marrow-derived suppressor cells [59].

### 2.8. Liver Ischemia-Reperfusion Injury

Liver ischemia-reperfusion injury mainly occurs during liver surgeries or transplantations, where the reduction of oxygen free radicals during the ischemia-reperfusion process decreases the endogenous antioxidant system, leading to liver damage. In a rat model of liver ischemia-reperfusion injury, CBD significantly reduced the expression of nitric oxide synthase, cyclooxygenase-2 (COX-2), NF-κB, Fas ligand, and caspase-3, while increasing survivin protein expression to protect the liver from ischemia-reperfusion injury [60]. Also, in a mouse model of liver ischemia-reperfusion, CBD markedly alleviated liver inflammation, oxidative stress, and cell death; this effect was independent of classical CB1/2 receptors [61].

### 2.9. Hepatic Glucose Metabolism and Diabetes

CBD’s beneficial effects on the livers of diabetic animals have been emphasized in various studies. In animal models, CBD’s anti-inflammatory and antioxidant properties indicate its potential as an anti-diabetic agent [62,63]. CBD significantly reduces the incidence of diabetes in mice, attributed to its substantial reduction of destructive insulin inflammation and inflammatory cytokines produced by the pancreas [64]. A study in rats on an HFD showed that intraperitoneal injection of CBD influenced sphingolipid metabolism, enhancing insulin sensitivity in subcutaneous and visceral fat tissues. This suggests CBD is a potential target for mitigating insulin resistance, type 2 diabetes, and metabolic syndrome [65].

The metabolic dysregulation observed in obese mice, linked to the activation of liver CB1 receptors [66], is counteracted by CBD, which exhibits antagonistic effects on CB1 receptors in the liver. In a study with a high-fat cholesterol diet mice model, CBD reduced blood glucose levels in a two-hour oral glucose tolerance test [22]. Further investigation in clinical trials of CBD is needed, but unfortunately, CBD’s intervention in type 2 diabetes patients did not show improvements in blood glucose and lipid parameters [67].

## 3. Molecular Pathways through which CBD May Affect Liver Disease

### 3.1. Cannabinoid Receptor 1

CB1 is prominently expressed in the central nervous system, with minor expression in adipose tissue, the liver, and skeletal muscle. CB1 is located in liver sinusoidal cells (LSECs), hepatic stellate cells, and hepatocytes. The expression of CB1 receptors in the liver is upregulated in viral hepatitis, cirrhosis, and alcoholic and non-alcoholic fatty liver diseases, while under normal conditions, it is diminished. CB1 plays a crucial role in the normal development of the liver; studies have found that the absence of CB1 and CB2 in zebrafish results in a smaller liver, reduced hepatocyte numbers, and impaired liver differentiation [68].

Most past research suggests that the absence or antagonism of CB1 can improve metabolic status, while CB1 overexpression is associated with the occurrence of metabolic diseases. This study confirms that inhibiting CB1 receptors can improve lipid synthesis in an MASLD model [69]. Exosomes produced by hepatocytes during lipotoxic injury play a role in the pathogenesis of MASLD, and plasma-derived exosomes from MASLD patients are carriers of CB1R transport, capable of regulating CB1 receptor expression in HepaRG cells [70]. Meanwhile, in Kupffer cells of mice fed HFD deficient in methionine choline, the expression of CB1 increases and is closely associated with the expression of the NLRP3 inflammasome [71]. The loss of CB1 in mouse liver or the application of CB1 antagonists can reduce hepatic steatosis and insulin resistance [72,73], decrease inflammasome activation, and improve inflammatory markers [74]. CB1 knockout in Kupffer cells shifts M1 pro-inflammatory to M2 anti-inflammatory, improving insulin signal transduction through Akt phosphorylation [75]. Liver-specific CB1 knockout in obese mice improves blood glucose through the liver SIRT1/mTORC2/Akt pathway and increases fatty acid oxidation through LKB1/AMPK [76]. CB1 activation in the liver leads to hyperleptinemia and leptin resistance [77]. Meanwhile, studies from cannabis smokers have shown other results [78]. CB1 receptors can be blocked by THC or CBD from cannabis. In addition, receptor antagonists reduced plasma leptin levels in obese individuals [79]. Thus, it might be expected CBD reduces leptin levels by blocking CB1 receptors, but other studies have found that leptin levels are significantly increased in cannabis smokers [80]. However, in some studies, there was no difference between cannabis smoking and blood leptin levels [81]. The conflicting findings may be due to differences in response based on gender and possible effects from endogenous cannabinoids.

Regarding liver fibrosis, CB1 promotes M1 macrophage polarization in mice through two independent pathways, RhoA/NF-κB and ERK1/2, leading to liver fibrosis [82]. However, a recent study reached the opposite conclusion, suggesting that liver-specific CB1 knockout did not prevent the development of MASLD and liver fibrosis in mice fed HFD [83].

CB1 is closely related to liver diseases caused by excessive alcohol consumption. In mice fed excessive alcohol and patients with alcohol-related liver cirrhosis, CB1 levels significantly increase [84,85]; at the same time, CB1 deficiency or the use of CB1 antagonists can inhibit HSC activation, significantly reducing alcohol-induced hepatic steatosis [86]. Also, blocking CB1 can lower blood lipids by reducing the secretion of low-density lipoproteins [87].

In individuals with chronic HCV infection and HCV-transfected liver cell in vitro models, the expression of CB1 in the liver significantly increases, and this increase is associated with disease progression [88]. In CON-A-induced acute liver injury, CB1 deficiency has an anti-inflammatory and anti-apoptotic effect by downregulating TNF-a and increasing NF-κB activation [89]. As a CB1 antagonist, rimonabant stimulates fatty acid β-oxidation and AMPK phosphorylation in rat muscle cells overexpressing GLUT4 [90].

CBD stabilizes the inactive conformation of CB1 or acts as a negative allosteric modulator of CB1, explaining its antagonistic effect. In vitro and in vivo studies suggest that CBD can antagonize agonist-induced CB1 activation. CBD allosterically reduces CB1 receptor signaling in HEK 293A cells [91]. CBD inhibits the reuptake and hydrolysis of anandamide, the most important endogenous CB1 receptor agonist, and exhibits neuroprotective antioxidant activity. Although CBD has a therapeutic effect on MASLD, alcoholic liver disease, viral hepatitis, and liver fibrosis, further research is needed to determine whether CBD’s antagonistic effect on CB1 can improve its adverse impact on the liver. Unlike endogenous cannabinoids, CBD’s antagonism of CB1 receptors and its ability to limit brain penetration make it a promising therapeutic agent for liver diseases [6].

### 3.2. Cannabinoid Receptor 2

CB2 is highly expressed in immune cells, mainly Kupffer and hepatic stellate cells [92]. The role of CB2 in MASLD is currently controversial, but previous studies have supported a role for CB2 in MASLD progression. For example, treating mice fed an HFD with CB2 agonists enhanced hepatic steatosis and insulin resistance [93], while CB2 deficiency in the liver could alleviate HFD-induced hepatic inflammation and improve insulin resistance [94]. In cultured human liver cell lines, the activation of CB2 induced an increase in CB1 expression, enhancing the negative effects of CB1 in the liver [95]. However, some studies present contrasting results, indicating that CB2 can reduce liver inflammation and fibrosis through various mechanisms. β-caryophyllene mediates AMPK and ACC phosphorylation via a CB2-dependent calcium signaling pathway, improving non-alcoholic fatty liver [96] and attenuating the pro-inflammatory M1 phenotype in Kupffer cells [97]. A recent study demonstrated that lignans with anti-fibrotic effects also act on CB2 receptors, inhibiting the NF-κB and p38MAPK pathways in Kupffer cells, thus improving CCl_4_-induced liver fibrosis [98].

In alcoholic liver disease, CB2 exhibits a protective effect in the liver in stark contrast to CB1. Depending on the different liver immune microenvironments, Kupffer cells differentiate into pro-inflammatory M1 phenotypes under the influence of alcohol. CB2 agonists induce the differentiation of Kupffer cells into anti-inflammatory M2 phenotypes responsible for tissue repair [92]. Additionally, CB2 can inhibit alcohol-induced liver inflammation by promoting liver autophagy [99]. In a mouse model of alcoholic liver disease, the absence of liver CB2 exacerbates hepatic steatosis, accompanied by increased HSC activation and collagen deposition [100].

In various other liver diseases, in a mouse model of ConA-induced liver injury, CB2 agonists alleviate liver inflammation by regulating CB2 receptors on Kupffer cells [101]. Necrotic inflammation induced by HCV infection is related to CB2 receptor dysfunction [102,103]. Activation of CB2 in the liver improves bile duct ligation-induced liver fibrosis and promotes liver regeneration [7]. CB2 also can inhibit the progression of HCC, and patients with high CB2 expression in HCC have a higher survival rate [8].

CBD has a low affinity for CB2 receptors. In Rheumatoid Arthritis Fibroblast-like Synoviocytes (RA-FLS), the activation of the CB2 receptor by CBD can also be observed [104]. Whether CBD, as a CB2 agonist, could alleviate liver inflammation by activating CB2 warrants further exploration. Controversially, in this study, CBD as an antagonist of CB2 receptor decreases the potency of the receptor agonist, WIN55212, in a GTPγS assay with membranes from CHO cells overexpressing CB2 receptors [105] Additionally, CBD can indirectly interfere with receptor activity by increasing endogenous ligands for CB1 and CB2, such as anandamide (AEA) and 2-arachidonoylglycerol (2-AG), which are arachidonic acid (AA). More research is needed to understand the role of CBD in the endocannabinoid receptors in the liver.

### 3.3. Peroxisome Proliferator-Activated Receptor

PPARs are critical transcription factors activated by various synthetic and endogenous ligands, participating in the regulation of multiple metabolic diseases. They play significant roles in lipid and carbohydrate metabolism in the liver while regulating energy metabolism, inflammation, and fibrosis. PPARs include PPARα, PPARβ/δ, and PPARγ, exhibiting differential distribution and functions across various liver cells.

PPARα is predominantly found in hepatocytes, hepatic fatty acid metabolism, and ketogenesis [106]. Mice with hepatic PPARα deficiency exhibited more severe liver inflammation and steatosis when fed HFD [107,108]. PPARα can reduce liver inflammation by disrupting the IL-6 signaling pathway [109]. Additionally, it can decrease liver fibrosis by activating hepatic peroxisomes or inhibiting stellate cell activation [110,111]. In a recent study, a newly discovered phytocannabinoid (E)-β-caryophyllene was found to counteract the reduction of PPARα in hepatic steatosis [112].

PPARβ/δ is distributed in hepatocytes, endothelial cells, and Kupffer cells [113]. Its agonists can lower blood glucose and improve insulin resistance [114,115]. Mice with hepatic PPARβ/δ deficiency exhibited disruptions in carbohydrate and lipoprotein metabolism [116]. 

PPARγ is mainly distributed in adipose tissue, with less distribution in the liver, so the effects on the liver are somewhat contradictory. It improves inflammation, oxidative stress, and fibrosis [117,118]. Hepatic PPARγ elimination worsened hyperlipidemia and insulin resistance in mice [119]. However, another study found that in obese patients and a mouse model of type 2 diabetes, PPARγ is upregulated [120,121,122].

As we saw earlier when we looked at the relationship between PPARs and liver disease, the application of CBD restored the protein expression of hepatic PPARα in a chronic alcohol-fed mouse model, improving alcohol-induced hepatic metabolic imbalance and steatosis [9]. Furthermore, this study found that CB1 co-evolved with PPARα, and blocking CB1 did not ameliorate hepatic steatosis in mice with hepatic PPARα deficiency [123]. CBD can activate PPARγ and influence its expression in vivo [124]. As an agonist of PPARγ, CBD induces reactive gliosis in rat primary astroglial cultures; this is significantly blunted by a selective antagonist of PPARγ receptors, GW9662 [125]. In mice models of skin fibrosis, it has also been reported that CBD upregulates the activity of PPARγ [126]. However, whether CBD has a similar impact on the activation of PPARγ in liver tissue remains to be determined.

### 3.4. G Protein-Coupled Receptor 55

GPR55, involved in inflammatory and energy metabolism processes, is expressed in rodents and human livers, yet its signaling pathway remains elusive [127]. The debate persists over the role of GPR55 in metabolic regulation. Some studies posit GPR55 as a promising anti-diabetic target, positively influencing β cell function [128]. In mouse models lacking GPR55 in the liver, there is a diminished insulin transmission in skeletal muscle and adipose tissue, resulting in induced obesity [129,130]. This research suggests that GPR55 agonists may safeguard β-cell apoptosis induced by endoplasmic reticulum stress by activating CREB [131]. However, conflicting findings indicate an upregulation of GPR55 in the livers of NASH patients. Knocking down GPR55 in vivo effectively ameliorates liver damage and methionine-choline deficiency in mice subjected to HFD. L-α-lysophosphatidylinositol (LPI), the sole known endogenous ligand for GPR55, stimulates GPR55 and ACC, promoting the activation of hepatic stellate cells [132]. Notably, LPI/GPR55 positively correlates with human obesity [133], and GPR55 agonists stimulate food intake, exacerbating obesity [134].

Some studies propose that GPR55 may enhance hepatic glucose and lipid metabolism when co-expressed with CB1 or CB2 [135,136,137]. Interestingly, the CB1 antagonist rimonabant acts as a GPR55 agonist under specific conditions. For instance, in mice lacking GPR55, the weight loss post-rimonabant treatment is less pronounced than in wild-type mice [138].

The role of CBD in GPR55 remains contentious. CBD, acting as an antagonist of the GPR55 receptor, modulates the levels of Ca^2+^ within neurons, thereby manifesting anti-convulsant characteristics [139]. While some studies depict CBD as a GPR55 antagonist, mitigating its pro-inflammatory effects, others suggest that abnormal cannabidiol (Abn-CBD), a CBD isomer, acts as a GPR55 agonist, reducing blood glucose and lipid levels in obese mice [140]. The precise mechanism by which CBD exerts its anti-inflammatory effects through GPR55 in the liver awaits further elucidation.

### 3.5. Transient Receptor Potential Channels

TRP channels, categorized into seven families as TRPC, TRPM, TRPA, TRPP, TRPM, and TRPN, play pivotal roles in sensory perception, vasodilation, cell apoptosis, and cell migration, thereby influencing the pathophysiological processes of cancer cells and immune cells [141,142,143,144]. Notably, these channels have been identified to express in liver cells, exerting a significant impact on regulating liver biological functions [145].

TRPV1^−/−^ mice show higher activation of hepatic stellate cells and severe liver fibrosis, while overexpression of TRPV1 has an anti-fibrotic effect. This study attributes the anti-liver fibrosis characteristics of TRPV1 to the prevention of hepatic stellate cell activation by recruiting SARM1 [146]. In addition, TRPV1 can also alleviate CCl_4_-induced liver fibrosis and weaken the effects of TGF- β on the activation, proliferation, and apoptosis of hepatic stellate cells, suggesting that the TRPV1 channel may be an effective treatment strategy for liver fibrosis [147]. TRPV1 affects cell plasticity by regulating Ovol2, Zeb1, and Sox10. The TRPV1 agonist capsaicin can inhibit the growth of HCC cells in xenograft models, making it a potential therapeutic target for human HCC [148]. Currently, there is controversy over the regulation of metabolic diseases by TRPV1. Some studies believe that TRPV1 plays a positive role in the metabolic process. TRPV1 alleviates T2DM-related liver injury by regulating OPA1 [149]. Regarding lipid regulation, TRPV1 agonists reduce lipid accumulation in the liver of wild-type mice and lower triglyceride levels. However, these effects are not seen in the liver of TRPV1 knockout mice [150]. Regarding glucose metabolism, the TRPV1 channel is an intrinsic component of the glucagon signaling pathway, and its absence is associated with increased glycogen production through PKA signaling [151]. Some studies have the opposite view. For example, this study found that TRPV1^−/−^ mice become obese, showing reduced physical activity, reduced energy consumption, aggravated liver steatosis, and reduced expression of thermogenic proteins in adipose tissue. The lack of TRPV1 does not prevent obesity but instead exacerbates metabolic dysfunction [152]. 

CBD’s interactions with liver TRP channels are primarily through TRPV1 and TRPV2. TRPV1 receptors are expressed in liver cells, vascular endothelial cells, and Kupffer cells, where they mediate acetaminophen (APAP)-induced liver damage, promote angiogenesis, and regulate Kupffer cell function [153,154]. In a mouse model causing experimental autoimmune hepatitis, CBD reduces pro-inflammatory factor levels in the liver, with the protective effect disappearing upon TRPV1 knockout [59]. Additionally, TRPV1 channels play a vital role in transmitting harmful stimuli. In the earlier literature, as a selective TRPV1 antagonist, capsazepine completely reversed the analgesic effect induced by CBD. These results indicate that the TRPV1 receptor remains the molecular target of CBD’s analgesic effect [155]. 

Some studies consider TRPV2 as a potential target for the treatment of human liver cancer patients. For example, the TRPV2 agonist probenecid and the TRPV2 antagonist tranilast significantly inhibit or promote the tumor growth of HepG2 xenografts in severe combined immunodeficiency (SCID) mouse models, respectively. In human HCC tissues, the expression level of the TRPV2 protein is inversely correlated with the expression levels of CD133 and CD44 proteins [156]. TRPV2 in the liver is currently known to mediate the survival of liver cancer cells. CBD’s ability to promote TRPV2 receptor activation enhances the anticancer effects of doxorubicin in liver cancer cell lines [11].

TRPA1 is a sensor for various harmful stimuli, including extreme cold, irritant compounds, and active chemical signals related to cell damage. CBD can serve as an agonist for TRPA1, and its potency is as high as 108% [157], compared to the well-known TRPA1 agonist, allyl isothiocyanate. However, there are currently few studies on the effects mediated by CBD on TRPA1. In mice on HFD supplemented with capsaicin (a TRPV1 agonist), menthol (a TRPM8 agonist), and cinnamaldehyde (a TRPA1 agonist), an increase in glucose utilization was observed, which prevented lipid accumulation and insulin resistance in the liver [10].

## 4. Liver Disease and CBD: Clinical Trials

### 4.1. MASLD and CBD

MASLD is the most prevalent liver disease worldwide, primarily caused by pathological processes such as triglyceride accumulation, fatty degeneration, and NASH. The pathogenesis of MASLD involves metabolic dysfunctions, including insulin resistance and lipid abnormalities, leading to hepatic steatosis, inflammation, and damage, ultimately resulting in fibrosis, cirrhosis, or even cancer.

Although CBD has been shown to have a specific therapeutic effect on MASLD in animal experiments, current clinical studies have not demonstrated a significant impact of CBD on MASLD. In an 8-week study involving patients with hepatic steatosis, daily doses of 200/400/800 mg CBD or placebo were administered, with no significant benefits observed in improving hepatic triglyceride levels [158]. Despite a study suggesting a reduced incidence of MASLD in cannabis users, the specific influence of CBD on MASLD remains unclear [159]. Clinical research on CBD in fatty liver disease is currently limited, and the results are not consistently positive, potentially due to factors such as treatment duration, CBD dosage, formulation, and insufficient sample size.

Furthermore, different formulations and routes of CBD administration can lead to absorption variations. CBD acts as an inhibitor of CYP450 enzymes. Although potential adverse interactions with other prescription drugs may occur, these events have been reported primarily in patients taking high doses of CBD.

The lack of precise pharmacokinetic and bioavailability data for CBD in the human body hinders its application in clinical trials [160]. There is still a need for more clinical studies to confirm the role of CBD in liver diseases, including determining the minimum effective dose and required plasma concentrations.

### 4.2. Cancer and CBD

HCC is a highly invasive and lethal cancer, accounting for over 85% of all malignant liver tumors. Current treatment options include surgical resection, liver transplantation, chemotherapy, and radiation therapy. However, these treatment methods have limitations, and there is an ongoing need to explore more effective therapeutic strategies to improve the prognosis of HCC patients. CBD has gained significant attention in cancer treatment. In in vitro and in vivo experiments with various human cancer cell lines, CBD has demonstrated inhibitory effects on cancer cell proliferation, migration, and angiogenesis [161]. As mentioned earlier, CBD promotes doxorubicin uptake in hepatocellular carcinoma (BNL1) cells by activating the TRPV2 channel, thereby facilitating doxorubicin-mediated cell death [11]. In a four-year study involving cancer patients using pharmaceutical-grade CBD, 92% of cases showed reduced circulating tumor cells or decreased tumor size [162]. This study suggests that intermittent administration of CBD is more effective than continuous administration, and the dosage of CBD is determined based on the mass of the tumor. The maximum dose is 30 mg, administered twice daily, and is gradually reduced after the condition stabilizes. The minimum duration of treatment required for CBD was six months. Despite the authors’ belief that no side effects of CBD were observed during the treatment, they should contemplate whether patients who discontinued prematurely due to adverse side effects within six months were excluded and whether the intricate clinical manifestations of advanced-stage cancer could have potentially obscured any adverse reactions to CBD.

CBD is widely used to alleviate pain, nausea, cachexia, and other adverse reactions in cancer patients [163]. It plays a role in relieving pain in late-stage cancer-related pain and opioid-resistant patients [164]. In a multicenter, randomized, placebo-controlled study, CBD demonstrated efficacy in managing symptom burden in advanced cancer patients receiving standard palliative care [165]. CBD may have certain benefits in treating tumors and can be recommended, but current clinical studies lack rigor. Future studies should pay greater attention to CBD’s pharmacokinetic properties, providing more detailed and tailored treatment plans that cater to specific demographics and diseases. Despite analyses based on large databases showing a lower incidence of hepatocellular carcinoma among cannabis users [36], there is still a lack of solid evidence for the clinical application of CBD in liver tumors.

### 4.3. Obesity and CBD

Obesity often leads to excessive fat accumulation in the liver and the occurrence of chronic inflammation, increasing the likelihood of severe liver diseases or HCC [166]. Previous studies suggested that CBD could promote fat cells’ browning and increase fat cells’ sensitivity to insulin [65,167]. Therefore, CBD is considered a potential treatment for preventing obesity. In animal obesity models, CBD attenuated skeletal muscle oxidative stress and inflammatory reactions caused by obesity, although it did not reduce body weight [168]. However, in this study, a derivative of cannabidiolic acid improved diet and gene-induced obesity and metabolic abnormalities [16]. Unfortunately, the beneficial effects of CBD on obesity have not been confirmed in current clinical studies. In a randomized, double-blind, placebo-controlled study, administering 100 mg of CBD twice daily for 13 weeks positively impacted insulin resistance and glucose-dependent insulinotropic peptide in non-insulin-treated type 2 diabetes patients. Still, triglyceride levels remained unchanged [67]. It appears that more research is needed to determine the impact of CBD on human obesity.

### 4.4. CBD Pharmacokinetics

The administration route, formulation, dosage of CBD, as well as dietary factors and gender all significantly influence the pharmacokinetics associated with CBD. Epidiolex^®^ (GW Pharmaceuticals, Cambridge, United Kingdom) is the only product that has undergone rigorous pharmacokinetic evaluation and clinical trial testing. It has earned approval from the U.S. Food and Drug Administration as a non-prescription drug for treating refractory epilepsy in children. However, research on CBD for other diseases is limited and often lacks the scientific rigor, controls, or sample size necessary to draw clinically meaningful conclusions. Presently, research on the pharmacokinetics of CBD is still in its infancy [4].

CBD is absorbed orally poorly and inconsistently, but co-ingestion with food can modify the metabolic kinetics of CBD. The absolute bioavailability of oral CBD under fasting conditions is approximately 6%, and when used with a high-fat diet, the absolute bioavailability of CBD increases fourfold [169,170].

The low oral bioavailability of CBD may be attributed to incomplete gastrointestinal absorption and the first-pass metabolism effect in the liver [169]. Therefore, inhalation or oral mucosal route minimizes the first-pass metabolism as much as possible. Inhalation can cause the plasma concentration of CBD to reach a rapid peak plasma concentration within 3–10 min, and the bioavailability is 11% to 45% [171,172].

Although transdermal administration of CBD can bypass hepatic first-pass metabolism, the hydrophobicity of CBD will cause it to be trapped in the aqueous layer of the skin before reaching blood circulation. Compared with oral or inhalation, the absorption rate of CBD through transdermal administration is lower [173].

The highest plasma CBD concentration can be achieved through intravenous injection. After intravenous injection of 20 mg deuterium-labeled CBD (2H2-CBD), the average blood drug concentration is 686 ng/mL 3 min after administration [160,174].

The half-life of CBD is 1.4~10.9 h after oral mucosal spray, 2~5 days after chronic oral administration, 24 h after intravenous injection, and 31 h after smoking. Compared with the oral/oral mucosal route, the area under the curve and Cmax are reached faster after smoking/inhalation, and Cmax increases in the dietary state and lipid formulation. Tmax is between 0 and 4 h [175].

A clinical study found that the oral formulation significantly affected the absorption of CBD. Epidiolex showed the highest Cmax, followed by oral capsules and syrup formulations [175]. In contrast, this randomized crossover study found no significant difference in plasma concentration between oral capsules containing CBD and CBD oil sublingual drops, which could result from CBD being swallowed before absorption in the mucosa [176].

In addition, gender differences may be another important source of inter-individual variation in CBD pharmacokinetics [177]. A double-blind and placebo-controlled crossover study showed that women absorb CBD more easily than men, possibly due to changes in body fat rate or hormones [178].

CBD is primarily excreted in feces and less in urine. The estimated plasma half-life is between 18 and 32 h [171]. The cytochrome P450 enzymes CYP3A4 and CYP2C9 in the liver hydroxylate CBD to 7-OH-CBD and 7-COOH-CBD. In healthy volunteers, the terminal elimination half-life after 750 and 1500 mg CBD 2 times/d is about 60 h [179]. The average half-life after intravenous administration is 24 ± 6 h, and after inhalation is 31 ± 4 h [180].

Since CBD acts on cytochrome P450 subtypes, it may cause interaction effects with other drugs, leading to an increase in the concentration of other drugs [181], which may, in turn, lead to adverse reactions to the drugs. CBD has many formulations, such as oil, sublingual tablets, capsules, sublingual sprays, nasal sprays, and creams, found in dietary supplements, cosmetics, and animal health products [182]. The recommended starting dose of Epidiolex is orally 2.5 mg/kg, twice a day, which can be doubled after a week (up to 10 mg/kg/day), twice a day (20 mg/kg/day) [183]. An earlier case report showed that long-term use of a high dose of 1500 mg/day did not produce adverse reactions in humans [184]. In this recent decentralized, observational study, adults from 18 to 75 years of age across the United States took CBD orally for a minimum of 30 days. They found self-medication of CBD did not appear to be associated with an increased prevalence of liver tests (LTs) elevation. Most LT elevations are likely due to the conditions/medications for which the individuals take CBD [185]. Caputi critically believes that the sample selection in this study lacks rigor. CBD users are disproportionately female (less likely to have liver toxicity) and exclude those who stopped use sooner due to adverse side effects within 30 days [186].

There is still a lack of research on the safety of long-term use of CBD, and we should pay attention to and prevent the potential hepatotoxicity risk caused by CBD. The impact of CBD on the liver usually occurs in the first few months of treatment, depending on the dose of CBD and the patient’s initial transaminase level [183]. CBD will hinder the liver metabolism of other drugs [187]; for example, in patients taking another antiepileptic drug with hepatotoxicity, valproic acid, the hepatotoxicity risk of concurrent use of CBD increases. In addition, the CBD AUC of patients with moderate or severe liver injury is 2.5–5.2 times higher than that of patients with normal liver function [179]. As CBD becomes increasingly popular in the consumer market, we need to understand better the safety risks associated with CBD.

## 5. Conclusions

**Figure 1:**
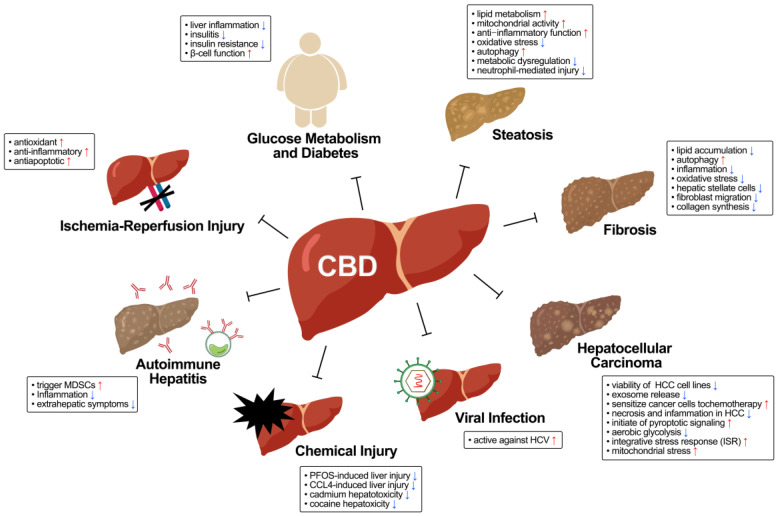
Overview of protective actions of CBD against liver diseases. The physiological effects of CBD in liver disease are depicted in the boxes. Red arrows represent increase, and blue arrows represent decrease.

The liver plays a crucial role in maintaining the homeostasis of the entire organism by metabolizing endogenous and exogenous substances. Liver diseases continue to pose a substantial global health burden, demanding the exploration of new and safe therapeutic strategies. CBD has been proven to benefit metabolic diseases in vitro, in vivo, and in clinical trials, revealing fresh potential in treating liver diseases (Figure 1). Understanding how CBD functions in diverse hepatic pathological environments is essential for pioneering innovative treatment approaches (Table 1). 

**Figure 2:**
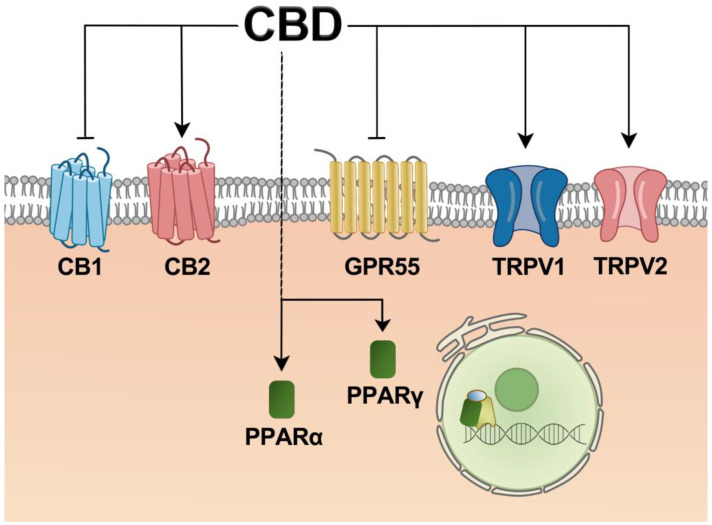
Schematic representation of potential receptor of CBD in the liver. Abbreviations: CBD, cannabidiol; CB1, cannabinoid receptor 1; CB2, cannabinoid receptor 2; GPR55, G protein-coupled receptor 55; TRPV1, transient receptor potential vanilloid channel 1; TRPV2, short receptor possible vanilloid channel 2; PPARα, peroxisome proliferator-activated receptor α; PPAR γ, peroxisome proliferator-activated receptor γ.

Currently, it is believed that CBD can interact directly with different receptors, thereby targeting multiple pathways and mechanisms. CBD can indirectly activate these receptors by regulating the concentration of endocannabinoids and related enzyme activities. Known receptors involved in CBD signaling include CB1, CB2, PPARs, TRPV1/2, and GPR55 (Figure 2). Table 1 shows that the effects identified in various cells may be consistent with animal studies, but not all studies have been equally validated. Furthermore, it is also not easy to replicate these effects in human clinical trials. As discussed above, CBD has been linked to various receptors expressed in vivo and physiological functions. Further research and clarification of the mechanisms involved in liver disease are required for effective commercialization. All these indicate that CBD has a vital regulatory function in the liver and is important in treating liver diseases.

Much work is still to be done, such as enhancing CBD’s pharmacokinetic and bioavailability data in the human body and conducting more extensive clinical trials based on this foundation.

## Figures and Tables

**Table 1:** A summary of CBD in liver disease.

Compounds	Model	Cell Lines/Animals	CBD Concentration	Mechanism	Ref.
CBD	Alcohol feeding-induced liver steatosis	C57BL/6J	5 or 10 mg/kg/day i.p. during the ethanol exposure 11 days	TNFα, MCP1, IL-1β, MIP2, E-Selectin mRNA, lipid peroxidation, 3-nitrotyrosine formation, NOX2, ACC-1, FASN protein ↓, PPARα, MCAD, ADIPOR-1, and mCPT-1 protein ↑	[9]
CBD	Co-treatment with doxorubicin on hepatocellular carcinoma	BNL1 ME cells	10 µM	TRPV2 channels ↑, P-glycoprotein ATPase transporter ↓	[11]
CBD	MASLD	HHL-5 cells, 3T3-L1 cells, Female ob/ob mice, Zebrafish	Female ob/ob mice:3 mg/kg/d oral gavage 4 weeks	CREB, ERK1/2, STAT2, STAT3, STAT6, AMPKa2, PRAS40 ↓	[12]
CBD	HFC diet, LPS + ATP ± CBD (MASLD)	RAW264.7 cells, C57BL/6J	5 mg/kg/d hand-fed 8 weeks	NF-κB-NLRP3 ↓	[13]
CBDA	HFD-fed mice, GIO mice	C57BL/6J, HepG2, HK-2 cells	20 or 40 mg/kg/d i.p. 28 days	obesity and its metabolic abnormalities ↓	[16]
CBD	Acute alcohol drinking-induced liver steatosis	C57BL/6, HepG2 cells	5 mg/kg/12 h i.p. 30 min before each ethanol gavage 5 days	JNK MAPK, autophagy ↑	[17]
CBD	Ethanol plus high-fat, high-cholesterol diet-induced liver steatosis	C57BL/6J	5 mg/kg/day gavaged 8 weeks	NF-κB–NLRP3–pyroptosis pathway ↓	[18]
CBD	High-fat/cholesterol diet (MASLD)	C57BL/6J	HFCD containing CBD (2.39 mg/kg)	TNF-α, iNOS ↓	[22]
CBD	CCl_4_-induced liver fibrosis	C57BL/6J	20 mg/kg/day oral gavage 2 weeks	TGF-β and IL-4 induced fibroblast migration ↓	[25]
CBD	PFOS-induced liver fibrosis	RAW264.7, AML12 and LX-2 cells, C57BL/6J	10 mg/kg gavage 30 days	CCDC25-ILK-NF-κB ↓	[26]
CBD	Liver Fibrosis	Hepatic stellate cells (HSCs)	5 µM 0–8 h	PERK-, ATF6-, and IRE1-mediated ER stress ↓, IRE1/ASK1/JNK pathway ↑ in activated HSCs	[29]
CBD	Hepatocellular carcinoma	HepG2 and ECACC cells	5 µM 60 min	Exosome and Micro-vesicle Release, CD63, STAT3 ↓	[41]
*C. sativa* extract	Diethylnitrosamine-induced hepatocellular carcinoma	Wistar rats	15 mg/kg or 30 mg/kg of C. sativa extract oral gavage 3 weeks	Akt, COX2, CRP, AFP mRNA ↓	[42]
CBD	Co-treatment with cabozantinib on hepatocellular carcinoma	HepG2 and Hep3B cells	<100 µM 48 h	P53, ER stress ↓	[45]
CBD	HCV/HBV infection	HepG2 2.2.15 cells	10 µM 6 days	HCV replication ↓	[53]
CBD	Cadmium hepatotoxicity	Rats	5 mg/kg/day i.p. 5 days	TNF-α, COX-2, NF-kB, caspase-3, and caspase-9 ↓, endothelial nitric oxide synthase↑	[55]
CBD	Cocaine-induced acute Hepatic Toxicity	Swiss mice	30 mg/kg/day i.p.	Reduced serum ALT and cardio green levels, inflammatory injury ↓	[56]
*C. sativa* extract	Oxidative-mediated hepatic injury	Albino rats	15–240 μg/mL	GSH level, SOD, and catalase activities↓, MDA and NO levels ↑, hepatic amylase and lipase activities, hepatic cholesterol and LDL-C, gluconeogenic activity ↓	[57]
CBD	Concanavalin A induced acute hepatitis	C57BL/6	5–50 mg/kg i.p. 6, 12, 24 and 48 h	TRPV1, Myeloid-derived suppressor cells (MDSCs) ↑	[59]
CBD	Ischemia/reperfusion (I/R)-induced liver injury	Rats	5 mg/kg/24 h i.v. (tail vein) 2 days	Nitric oxide synthase, COX-2, NF-κB, Fas ligand and caspase-3 ↓, Surviving protein ↑	[60]
CBD	Ischemia/reperfusion (I/R)-induced liver injury	C57BL/6J	3 or 10 mg/kg i.p. 60 min before the occlusion of the hepatic artery and the portal vein Or i.v. (femoral vein) before the reocclusion or 90 min after	Oxidative/nitrative stress, NF-κB, TNF-α production in isolated Kupffer cells ↓, adhesion molecule expression ↓ independent of CB1/2 receptors	[61]

↑, increase; ↓, decrease.

## Data Availability

No new data were created or analyzed in this study. Data sharing is not applicable to this article.

## Abbreviations

CBD	Cannabidiol
MASLD	Metabolic dysfunction-associated steatotic liver disease
NAFLD	non-alcoholic fatty liver disease
CB1	cannabinoid receptor 1
CB2	cannabinoid receptor 2
PPARs	peroxisome proliferator-activated receptors
TRPs	transient receptor potential channels
HCC	hepatocellular carcinoma
HFD	high-fat diet
TGF-β	transforming growth factor-β
PPAR γ	peroxisome proliferator-activated receptor γ
PPARα	peroxisome proliferator-activated receptor α
NF-κB	nuclear factor kappa-light-chain-enhancer of activated B cells
AMPK	5′AMP-activated protein kinase
ACC	Acetyl-coenzyme A carboxylase
NLRP3	NLR family pyrin domain containing 3
TRPV1	Transient receptor potential vanilloid 1
TRPV2	Transient receptor potential vanilloid 2
TRPA1	Transient receptor potential ankyrin 1
TRPM8	Transient receptor potential melastatin 8
GPR55	G protein-coupled receptor 55
2H2-CBD	deuterium-labelled cannabidiol
HSCs	hepatic stellate cells
GSDME	gasdermin E
COX-2	cyclooxygenase-2
LSECs	liver sinusoidal cells
AEA	anandamide
2-AG	2-arachidonoylglycerol
AA	arachidonic acid
LPI	L-α-lysophosphatidylinositol
Abn-CBD	abnormal cannabidiol
APAP	acetaminophen
NASH	non-alcoholic steatohepatitis
RA-FLS	Rheumatoid Arthritis Fibroblast-like Synoviocytes
LTs	liver tests
